# Digital Remote Phase-II Cardiac Rehabilitation After Valvular Surgery: A Quasi-Experimental Study

**DOI:** 10.1016/j.jacasi.2026.07.005

**Published:** 2026-08-20

**Authors:** Mariya P. Jiandani, Vijaya Bhurke, Ayesha Shaikh, Chetan Gharat

**Affiliations:** a Physiotherapy School and Centre, Seth GS Medical College and KEM Hospital, Mumbai, Maharashtra, India; b Medical Affairs, Lupin Digital Health Limited, Mumbai, Maharashtra, India

**Keywords:** 6-minute walk test, EuroQol visual analogue scale score, quality of life, software-driven remote cardiac rehabilitation, valvular heart disease

Valvular heart disease (VHD) is a major cause of morbidity, mortality, and impaired quality of life (QoL),^1^ particularly in the Asia-Pacific region.^2^ In India, rheumatic heart disease affects approximately 54.8 million individuals, and calcific aortic valve disease and degenerative mitral valve disease affect 13.3 and 15.5 million individuals, respectively.^3^ Although valve surgery improves survival and symptoms,^4^ postoperative recovery remains challenging,^5^ highlighting the need for structured cardiac rehabilitation (CR).

Clinical guidelines recommend CR after valve surgery,^5^ yet its use remains low because of limited access, low referral rates, lack of awareness, and poor adherence to center-based programs.^6^ Remote cardiac rehabilitation (RCR) offers a potential solution by delivering exercise guidance, medication education, nutritional counseling, and psychological support via digital platforms.^6^ However, research for RCR in post–valvular surgery patients is limited.

This study evaluated the feasibility and effectiveness of a remote, software-based phase-2 CR program on functional capacity and health-related QoL after valvular heart surgery. A single-center, parallel-group, quasi-experimental comparative study was conducted over 12 weeks at a cardiovascular and thoracic surgery outpatient department of a tertiary care hospital. The clinical study protocol (CTRI/2024/06/068,230) was approved by the institutional ethics committees (approval number: EC/31/2024) and conducted in accordance with Good Clinical Practice Guidelines and the Declaration of Helsinki. Written informed consent was obtained from each participant before enrollment in the study.

Adults aged 18 to 60 years undergoing elective left- or right-sided valvular surgery, including double-valve or combined valve and coronary artery bypass graft procedures, were included. Exclusion criteria from the study included cognitive impairments, left ventricular dysfunction, or a history of persistent or paroxysmal atrial fibrillation, unstable postoperative hemodynamic status, lower limb deformities impairing ambulation, or refusal to provide consent to participation.

The Remote Phase-II CR program was a structured 12-week intervention based on the FITT (Frequency, Intensity, Time, Type) principle. The program included individualized exercise prescription, education sessions, relaxation training, medication adherence monitoring, and nutritional counseling reinforcement. Participants were assigned to group A (software-driven digital intervention for smartphone users) or group B (usual care). In group A, education and relaxation content were delivered via an app, with Google Fit used for step tracking, and adherence monitored through weekly videoconferencing and telephonic consultations. Group B received standard postoperative care with general advice on activity, diet, medications, and follow-up, without a structured or supervised exercise program. The exercise plan was designed based on baseline functional capacity assessed by the 6-minute walk test and the patient's clinical condition, to assess functional capacity, performed routinely as part of standard care before discharge after cardiac surgery. The test was conducted along a flat, straight 30-m corridor, with the starting line marked using brightly colored tape and turnaround points indicated by a cone. Primary outcomes were changes in 6-minute walk distance (6MWD) and EuroQol 5-dimensional questionnaire (EQ-5D) QoL scores over 12 weeks, with secondary outcomes including exercise and medication adherence and comfort with the software-driven Phase-II CR program.

Data were summarized as mean, SD, or counts and percentages, and group comparisons were made using Student's *t*-tests, paired samples *t*-tests, and repeated measures analysis of variance (ANOVA). Of 60 participants, 58 completed the study and were divided into group A (software-driven intervention, n = 28) and group B (usual care, n = 30). Two-factor repeated measures ANOVA was used with time (baseline, 12 weeks) as the within-subject factor and group (A, B) to assess the time-by-group interaction.

Baseline demographics and clinical characteristics were comparable between groups, with mean ages of 46.18 (10.14) years and 41.53 (9.71) years, body mass index of 23.18 (3.99) kg/m^2^ and 22.51 (3.78) kg/m^2^, and similar sex distribution; comorbidities were largely similar except for higher diabetes and thyroid disorders only in group B. The 76.67% (23 of 30) of group B patients underwent mitral valve replacement, followed by tricuspid and double valve replacement (16.67% [5 of 30] participants each).

At 12 weeks, both groups reported significant improvements in functional capacity and QoL, with group A demonstrating greater gains. The mean change in 6MWD was 243.17 minutes (95% CI: 217.56-268.78; *P* < 0.001) in group A vs 112.15 minutes (95% CI: 97.22-127.08; *P* < 0.001) in group B, indicating nearly double the improvement with the digital application. Similarly, both EQ-5D utility and visual analogue scale scores showed significant enhancements in perceived health status and QoL, again with greater improvements in group A. These differences were statistically significant as confirmed by repeated measures ANOVA, with F-values for 6MWD, EQ-5D utility, and visual analogue scale all significant at *P* < 0.001 (Figure 1). These findings are consistent with previous studies. A randomized controlled trial demonstrated that CR programs significantly improved 6-minute walk test outcomes within 6 months post valvular surgery.^4^ Another study reported a 13% improvement in functional capacity, from 349 to 393 minutes (*P* = 0.0016), after CR in a similar population.^7^ International research supports the effectiveness of remote CR, programs like Physical Activity Towards Health demonstrate improved adherence, whereas home-based CR trials show improved QoL and cost-effectiveness in patients with heart failure.^8^^,^^9^

**Figure 1:**
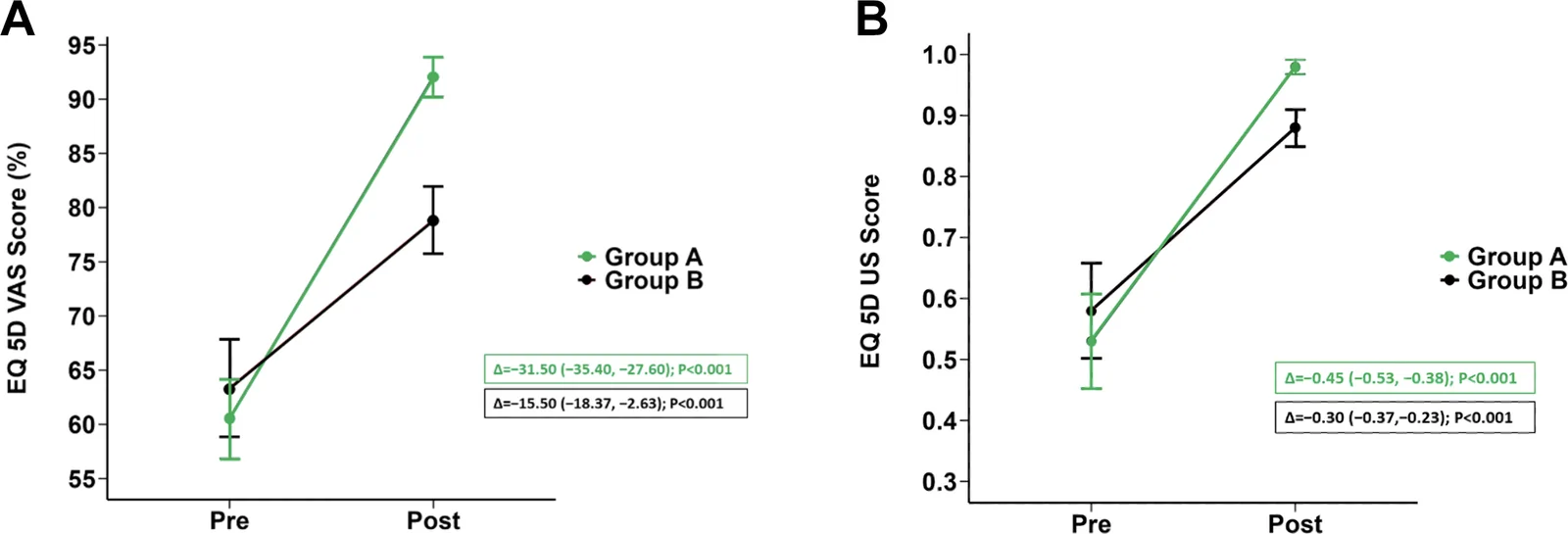
Changes in EQ-5D VAS and EQ-5D Utility Scores (A) Illustrated is the change in health-related quality of life measured by the EQ-5D VAS before (Pre) and after (Post) intervention in group A and group B. Mean scores with error bars (± SD) are shown. Both groups demonstrated significant improvement, with group A showing a greater mean increase (Δ = 31.50) compared with group B (Δ = 15.50; both *P* < 0.001). These findings suggest superior improvement in perceived overall health status in group A following intervention. (B) Presented is the change in EQ-5D utility scores from Pre to Post intervention in group A and group B. Mean values are displayed with error bars (± SD). Both groups showed statistically significant improvement (*P* < 0.001), with group A demonstrating a larger mean gain (Δ = 0.30) compared with group B (Δ = 0.45). The results indicate clinically meaningful improvement in health utility following treatment, with a differential magnitude between groups. Green circles represent group A and black circles represent group B. EQ-5D US = EuroQol 5-dimensional questionnaire utility score; EQ-5D VAS score = EuroQol visual analogue scale score.

Secondary outcomes further supported the intervention's effectiveness, with higher medication adherence in group A at both 6 and 12 weeks. Physical activity adherence was markedly better in group A. Average physical activity increased by 89.6% from 230.00 (81.85) minutes/week to 435.89 (172.41) minutes/week in group A, compared with a 53.7% increase from 206.67 (63.22) minutes/week to 317.67 (78.82) minutes/week in group B. All 100% (28 of 28) of group A participants exercised at least 3 times/week, compared with only 6.67% (2 of 30) of participants in group B.

Acceptability and feasibility of the software-based program were high. All participants in group A strongly agreed that RCR was an acceptable mode of delivery, whereas 90% (27 of 30) of group B participants disagreed. This difference suggests that the structured and engaging nature of the digital intervention may have promoted higher motivation, satisfaction, and perceived utility.

This is the first Indian study evaluating a digital, application-driven RCR program in patients with VHD. Digital interventions are feasible and may outperform conventional care in improving outcomes, self-management, and satisfaction in Indian patients with VHD, addressing key evidence gaps in resource-limited settings.

Limitations include the requirement for smartphone access and digital literacy, which may introduce selection bias, and a short follow-up period. Secondary outcomes were exploratory and not powered for statistical testing, limiting interpretability. Large randomized controlled trials with longer follow-up are needed.

In conclusion, a software-driven RCR program improved functional capacity, QoL, medication adherence, and physical activity, supporting its role in expanding CR access.

## Funding Support and Author Disclosures

Drs Gharat and Shaikh are employed by Lupin Digital Health, the developer of the digital CR program LYFE application, which is evaluated in this study. Drs Jiandani and Bhurke are employed by Seth GSMC and KEMH.

## References

[1] Santangelo G., Bursi F., Faggiano A. (2023). The global burden of valvular heart disease: from clinical epidemiology to management. J Clin Med.

[2] So K.C., Yap J., Song G.Y. (2025). Epidemiology of valvular heart disease in Asia Pacific Region. JACC Asia.

[3] Chen Q.F., Shi S., Wang Y.F. (2024). Global, regional, and national burden of valvular heart disease, 1990 to 2021. J Am Heart Assoc.

[4] Xue W., Xinlan Z., Xiaoyan Z. (2022). Effectiveness of early cardiac rehabilitation in patients with heart valve surgery: a randomized, controlled trial. J Int Med Res.

[5] Butchart E.G., Gohlke-Bärwolf C., Antunes M.J. (2005). Recommendations for the management of patients after heart valve surgery. Eur Heart J.

[6] Itoh H., Amiya E., Jimba T. (2024). Efficacy and safety of remote cardiac rehabilitation in the recovery phase of cardiovascular diseases (RecRCR study): a multicenter, nonrandomized, and interventional trial in Japan. Int J Cardiol Heart Vasc.

[7] Pollmann A.G.E., Frederiksen M., Prescott E. (2017). Cardiac rehabilitation after heart valve surgery: improvement in exercise capacity and morbidity. J Cardiopulm Rehabil Prev.

[8] Claes J., Cornelissen V., McDermott C. (2020). Feasibility, acceptability, and clinical effectiveness of a technology-enabled cardiac rehabilitation platform (physical activity toward health-I): randomized controlled trial. J Med Internet Res.

[9] Dalal H.M., Taylor R.S., Jolly K. (2019). The effects and costs of home-based rehabilitation for heart failure with reduced ejection fraction: the REACH-HF multicentre randomized controlled trial. Eur J Prev Cardiol.

